# Predator-Driven Nutrient Recycling in California Stream Ecosystems

**DOI:** 10.1371/journal.pone.0058542

**Published:** 2013-03-08

**Authors:** Robin G. Munshaw, Wendy J. Palen, Danielle M. Courcelles, Jacques C. Finlay

**Affiliations:** 1 Earth to Ocean Research Group, Department of Biological Sciences, Simon Fraser University, Burnaby, British Columbia, Canada; 2 Department of Ecology, Evolution and Behavior, University of Minnesota, St. Paul, Minnesota, Unites States of America; University of California, Berkeley, United States of America

## Abstract

Nutrient recycling by consumers in streams can influence ecosystem nutrient availability and the assemblage and growth of photoautotrophs. Stream fishes can play a large role in nutrient recycling, but contributions by other vertebrates to overall recycling rates remain poorly studied. In tributaries of the Pacific Northwest, coastal giant salamanders (*Dicamptodon tenebrosus*) occur at high densities alongside steelhead trout (*Oncorhynchus mykiss*) and are top aquatic predators. We surveyed the density and body size distributions of *D. tenebrosus* and *O. mykiss* in a California tributary stream, combined with a field study to determine mass-specific excretion rates of ammonium (N) and total dissolved phosphorus (P) for *D. tenebrosus*. We estimated *O. mykiss* excretion rates (N, P) by bioenergetics using field-collected data on the nutrient composition of *O. mykiss* diets from the same system. Despite lower abundance, *D. tenebrosus* biomass was 2.5 times higher than *O. mykiss*. Mass-specific excretion summed over 170 m of stream revealed that *O. mykiss* recycle 1.7 times more N, and 1.2 times more P than *D. tenebrosus*, and had a higher N:P ratio (8.7) than that of *D. tenebrosus* (6.0), or the two species combined (7.5). Through simulated trade-offs in biomass, we estimate that shifts from salamander biomass toward fish biomass have the potential to ease nutrient limitation in forested tributary streams. These results suggest that natural and anthropogenic heterogeneity in the relative abundance of these vertebrates and variation in the uptake rates across river networks can affect broad-scale patterns of nutrient limitation.

## Introduction

The productivity of primary producers in aquatic ecosystems is limited by a range of biotic and abiotic factors, with light and nutrient availability among the primary drivers [1]–[6]. Nutrient limitation can over-ride the importance of light, and even in low-light environments, increasing the availability of nutrients can increase primary producer biomass [7]. Nitrogen (N) and phosphorus (P) are key macronutrients that can individually or simultaneously limit growth depending on their availability and the requirements of the demand assemblage [1], [8]. External ambient nutrient fluxes of N and P are driven by weathering, fixation, and runoff from terrestrial ecosystems [9] as organic and inorganic components of soils release dissolved mobile ions in surface waters [10]. In addition, internal biotic processes can subsidize fluxes into the system, increasing nutrient availability.

In aquatic environments, animals process organic compounds through consumption, metabolism, and excretion into labile dissolved forms that can be taken up by autotrophs and other microbial organisms [11]. This process of consumer-driven nutrient recycling subsidizes ambient nutrient levels, and can affect the community structure of phytoplankton in lakes [12], [13], control the availability of nutrients in phytotelmata [14], and control the spatial distribution of nutrients in river systems [15]. The rate at which nutrients are recycled within a system depends heavily on the physical characteristics of that system and characteristics of the organisms that inhabit it.

Interactions among producers, herbivores, and predators are important determinants of nutrient dynamics in aquatic ecosystems. Changes in nutrient availability can have direct effects on producer biomass [16], as well as potential for future growth through nutrient storage [17]. As the base of the food web, producers in streams can drive ecosystem productivity and can be directly and indirectly affected by a range of animals. Herbivores can directly limit producer growth and biomass through grazing pressure [18]. Higher trophic level animals indirectly affect autotroph abundance by stimulating higher producer productivity through nutrient recycling and preying on herbivores, which in turn reduces grazing pressure [13], [19]–[22].

To date, investigations of the effects of consumer identity and abundance on nutrient recycling in streams have focused mainly on fish assemblages (e.g. [12], [15], [23]–[30], but see [31]–[34]). While fish species represent a large fraction of animal biomass in many stream ecosystems, there are myriad other taxa that make up a substantial component of secondary production, including aquatic macroinvertebrates and other vertebrates (amphibians, reptiles, mammals, birds). Predatory amphibian populations in particular, often rival the abundance and biomass of fishes in many systems, and may have similarly large effects on nutrient recycling. While some previous work has estimated the potential for nutrient recycling by a limited number of amphibians at broad scales [11], [35], excretion data for salamanders is non-existent for most species, and they have seldom been considered as potentially important nutrient recyclers [36]. In temperate tributary streams such as those in the Pacific Northwest, amphibian populations can reach extremely high densities [37]–[39], even surpassing those of fishes [40], [41]. In this region, two main vertebrate groups dominate predator assemblages; larval salamanders of the Genus *Dicamptodon* and juvenile salmonids of the Genus *Oncorhynchus*. Larval salamanders can often occur at densities exceeding all other predators, accounting for up to 99% of the vertebrate biomass in some areas [42]. *Dicamptodon tenebrosus* (coastal giant salamander; [43]) is one of the largest salamanders in North America, and often co-occurs with *Oncorhynchus mykiss* (steelhead trout). Both vertebrates are similarly opportunistic predators [44], [45]. As large bodied vertebrates, these predators have may fill similar ecological niches and may contribute similarly to ecosystem processes such as nutrient recycling.

Here we examined the effects of species identity in nutrient recycling between two taxonomically diverse top-predators, *D. tenebrosus* and *O. mykiss*, and explored the relative importance of ecosystem-scale N and P recycling by these predators across a range of potential real-world densities. We conducted a field study to estimate diet composition, nutrient excretion rates (N, P), and densities for each species, as well as the elemental body composition (C, N, P) of their prey in a coastal stream in Northern California. We hypothesize that, due to their large body size and high abundance [42], *D. tenebrosus* would dominate predator-mediated nutrient recycling in tributary ecosystems where both species co-occur. We also hypothesize that changes in the relative abundance of each species could affect ecosystem-level nutrient availability.

## Methods

To quantify the magnitude and importance of nitrogen and phosphorus recycling by vertebrates in tributary biogeochemistry, we conducted a survey of the size distribution, abundance, and diet composition of *D. tenebrosus* and *O. mykiss.* We estimated mass-specific excretion rates by two methods; *in situ* incubations for *D. tenebrosus* to establish novel excretion rate estimates, and bioenergetics modeling for *O. mykiss*. Bioenergetics models are often substituted for direct excretion measurements when appropriate models and parameter estimates exist [13], [46]–[49]. Using a bioenergetics model to estimate excretion rates has been shown to be a very equitable surrogate for direct measurement [50]. However, due to a lack of available data on *D. tenebrosus*, direct measurements were necessary to establish excretion estimates. Despite the uncertainties of using two methodologies for determining excretion rates, both methods can be considered comparable if adequate care is taken with handling and incubation time to minimize the effects of stress and fasting [11], [23], [51]. By using a bioenergetics model for fish, we were able to avoid the error in estimating normal excretion rates caused by handling stress, and reduce the need to experimentally manipulate additional animals.

Although aquatic vertebrates can produce similar daily quantities of N and P through egestion and excretion [49], nutrients in fecal matter must undergo further microbial processing before they are available for uptake by autotrophs. We therefore do not consider egestion as an instantaneous contribution to nutrient recycling in this study. As such, we acknowledge that our estimates of excretion represent underestimates of total nutrients recycled by vertebrates. We parameterized an *O. mykiss* bioenergetics model using surveyed diet composition, prey whole-body nutrient compositions (carbon, nitrogen, phosphorus), and summer water temperatures collected from several tributary streams. We combined estimates of *O. mykiss* and *D. tenebrosu*s population densities with mass-specific excretion rates to estimate the relative magnitude of nutrient recycling by each predator. Using these excretion estimates and literature values for local nutrient uptake rates, we determined total vertebrate contribution to nutrient demand, and lastly explore the consequences of different relative abundances of *D. tenebrosus* and *O. mykiss* for ecosystem level nutrient availability.

### Ethics Statement

This work was conducted with the approval California Department of Fish and Game (#11077), NOAA (#14904), and Simon Fraser University Animal Care (920B-09) permits. All animals were anaesthetized using Tricaine methanesulfonate (MS-222) before handling, and all efforts were made to minimize stress and suffering during this study.

### Study Site

Like many coastal tributaries in Northern California, Oregon, and Washington, our study site (Fox Creek, South Fork Eel river watershed, UTM: 10S 445880E, 4399070N) has two classes of vertebrate predators, stream salamanders (Genus *Dicamptodon*) and juvenile salmonid fishes (Genus *Oncorhynchus*). *Dicamptodon tenebrosus* exhibits life-history plasticity; after two to three years as aquatic juveniles, *D. tenebrosus* can either remain aquatic as paedomorphic adults or metamorphose into terrestrial adults [52]. The geographic range of *D. tenebrosus* includes coastal watersheds from the southern extremes of British Columbia to central California with the exception of the Olympic Peninsula [53]. *Oncorhynchus mykiss* populations in Pacific coastal watersheds are usually comprised of a mix of anadromous (i.e. steelhead) and resident (i.e. rainbow trout) individuals, with juveniles rearing in freshwaters for one to two years typically followed by divergence into one of the two dominant life-histories [54], [55]. To determine the mass-frequency distribution and total biomass represented by each species, we conducted a survey of *D. tenebrosus* and *O. mykiss* in a tributary typical of coastal watersheds in the Pacific Northwest. We conducted our survey on Fox Cr. (2.6 km^2^ drainage area), a perennial tributary of the South Fork Eel River (SF Eel), Mendocino County, California, whose watershed lies entirely within the University of California’s Angelo Coast Range Reserve. The Fox Cr. watershed is dominated by Douglas fir (*Pseudotsuga menziesii)*, Coast redwood (*Sequoia sempervirens)*, and mixed conifer-deciduous forests. The channel is moderately incised with steep banks and is heavily shaded, and frequent woody debris jams are indicative of high winter discharge (November-March) despite low dry-season base flow (April-October, 5–7 L⋅s^−1^; [45]). During summer base flow, the stream is reduced to a series of short riffles and shallow pools. The streambed alluvium is a mixture of cobbles and boulders, embedded with sand and pebbles.

### Abundance Survey

To estimate the body size distribution and total biomass of *D. tenebrosus* and *O. mykiss*, we surveyed all individuals of both species in a sub-set of pools within the first 1.3 km of Fox Cr. Both *O. mykiss* and *D. tenebrosus* co-occur in Fox Cr. from the confluence with the SF Eel to approximately 1.3 km upstream, at which point fish passage is restricted, and only *D. tenebrosus* is present. Based on previous surveys, these two species constitute the vast majority of vertebrate biomass in tributary streams in the region and are present year round [38]–[40], [56]–[58]. Animals were surveyed by serial depletion in 32 tributary reaches (total stream distance of 170 m) blocked with nets from the top of the upstream riffle to the downstream end of each pool, using a combination of methods including snorkeling, electro-shocking, and hand-capture using the Parker-stick method [45]. After capture, animals were anesthetized with MS-222, weighed (g), and measured for total length (TL), snout to vent length (SVL, for *D. tenebrosus*) and standard length (SL, for *O. mykiss*). We used SVL and SL measurements as the primary measures of body length due to the frequency of tail injuries that can bias TL measurements. To compare dietary intake of N and P, a subset of captured individuals were sampled for diet using non-lethal gastric lavage (*O. mykiss n* = 86, *D. tenebrosus n* = 55). We adopted a minimum size threshold of 100 mm SL for *O. mykiss* and 50 mm SVL for *D. tenebrosus* based on lavage apparatus to avoid injuring small individuals during diet collection. Individuals with extensive injuries were not dieted. All individuals in the diet survey were captured in the daytime in pools, controlling for diel and habitat variability in gut contents. Individuals were dieted opportunistically across several concurrent studies, which is reflected by the inconsistent sample sizes. Diets were preserved in 70% EtOH until enumeration, measurement, and identification to the highest level of taxonomic resolution possible (e.g. Genus or Family). Dry biomass of individual diet items was estimated using measured length (mm) and taxon-specific length-weight regressions developed from our survey data and the literature (Table S1). Diet data were supplemented with a longer-term data set from our study stream and nearby tributaries. Water content of prey items was determined from the literature [59], and used to convert invertebrate dry weight composition of C, N, and P to wet weight composition for use in the *O. mykiss* bioenergetics model. After processing and recovery from anesthesia, all animals were released live at the point of capture.

### Quantification of Nutrient Excretion

#### *D. tenebrosus* field study

To estimate nutrient-specific excretion rates and ratios for larval *D. tenebrosus*, we incubated 18 individuals (SVL range: 56–133 mm) with one of three common diet items [45], [60]; terrestrial invertebrates (Orthoptera adults), aquatic invertebrates (Odonata larvae), or aquatic vertebrates (young-of-the-year *O. mykiss*). Individuals for excretion trials were collected by hand from Fox Cr. and placed in window screen-covered flow-through buckets to prevent the introduction of additional prey. Minimum dietary throughput for *D. tenebrosus* in this system is estimated to be 60 hours (Munshaw *unpublished data*), therefore salamanders were incubated with food treatments for 60 hours (±2 hours) to allow excretion rates and ratios to reflect dietary treatments. Food treatments were assigned to control for unknown diet composition that may introduce additional variation into our estimates of excretion rates and ratios. To allow study salamanders to feed *ad libitum*, we kept each enclosure stocked with an excess of treatment diet items. This reflects the high availability of prey items in the ecosystem, and low occurrence of individuals with empty stomachs [45]. After 60 hours, salamanders were removed from flow-through enclosures, gently rinsed with filtered water to remove adhered particles, and immediately placed in individual 2 L acid-washed containers with 1 L of 0.7 µm-filtered (Whatman GF-F) stream water. Containers were covered with loose plastic lids to prevent addition of airborne particles, and incubated in the stream margins (approx. 5 cm depth) to maintain ambient stream temperature for the duration of the incubation. In addition to incubating salamanders to measure nutrient excretion, we also incubated 3 control containers without salamanders to evaluate the change in nutrient concentrations due to microbial processes, despite our care with filtration. After 120 minutes, approximately 100 mL of water was filtered (0.7 µm, Nalgene 190 syringe filter) to measure ammonium (NH_4_) and soluble reactive phosphorus (SRP), and animals were removed, weighed, measured as above and released. Water samples were kept dark in coolers with ice packs and were processed within 4 hours. SRP concentrations were determined using spectrophotometry, and ammonium concentrations were determined using fluorometry [61].

To evaluate the effects of mass and diet group on *D. tenebrosus* excretion rates, we compared the likelihood of several competing linear regression models using Akaike’s Information Criterion for small sample sizes (AICc). We fit linear models by maximum likelihood including all combinations of mass (*log_10_*(g)) and diet treatment (terrestrial invertebrate, aquatic invertebrate, aquatic vertebrate), including a mass by diet treatment interaction and an intercept only model, assuming normally distributed errors. We used the best-supported model by AICc to estimate daily excretion rates (N, P) for each individual from our survey reach and summed across all individuals (n = 348 *D. tenebrosus*) to estimate the total amount and ratio of N and P recycled by *D. tenebrosus* in the 1 km reach.

#### *O. mykiss* bioenergetics model

We estimated *O. mykiss* mass-specific excretion rates using an established bioenergetics model for salmonids [62]. This model was built using a range of body masses that fully encompass those found in our study. Nutrient specific (total dissolved N, total dissolved P) bioenergetics models estimate mass-specific excretion rates (*E_i_*) for each individual (*i*) in grams per day based on animal mass (*M_i_*), water temperature (*T*), and N and P content of diet items (*N*).
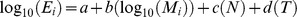



Schindler and Eby [62] determined coefficients (*a, b, c, d*) for a generalized salmonid bioenergetics model through meta-analysis and literature review including several species of salmonids, and we applied those estimates to our model (N: *a* = −3.256, 0.084 SE, *b = *0.893, 0.021 SE, *c* = −15.049, 5.526 SE, *d = *0.014, 0.003 SE, P: *a* = −4.776, 0.068 SE, *b = *0.902, 0.024 SE, *c = *96.801, 19.288 SE, *d = *0.008, 0.003 SE). Each time we applied the model to an individual, we selected coefficients from the error distribution associated with each estimate. To parameterize the remaining terms of our *O. mykiss* bioenergetics model, we surveyed the diet composition of *O. mykiss* (>50 mm TL) in Fox Cr. as described above. Diets were converted to biomass pooled by order (as described above), and the average C, N, and P composition for each order was estimated based on percent C, N, and P measured from invertebrates collected during a 2008 survey of tributary sites within the SF Eel watershed (J. Hood, *unpublished data*). Invertebrate C and N composition was determined using an elemental analyzer on dried and homogenized individuals or composited samples of multiple individuals. Samples for P were ashed at 550°C and hydrolyzed with HCl followed by colorimetric determination of PO_4_ [63]. Multiple values for the same genus were averaged, and values for genera not represented in the survey were summarized from the literature (Table 1 footnote). Diet biomass and nutrient compositions (C, N, and P) were converted to wet-mass using length to wet mass regressions for terrestrial and aquatic diet items separately [64]. We used the combination of the diet composition from surveyed fishes and invertebrate body composition to determine the nitrogen and phosphorus composition of an average steelhead diet, and used these values in the *O. mykiss* bioenergetics model. Hourly stream temperatures were recorded in 11 pools within the 1.3 km study reach using iButton temperature loggers (Maxim Integrated Products Inc., Sunnyvale, CA) in Fox Cr. for a 7-week period (July-August) in 2010 during the peak season of biological productivity. Average daily temperatures at 11 stream locations were averaged to calculate stream-wide mean temperature (*T*). We used the average dietary nitrogen and phosphorus values (*N, P*), along with individual weight (*M_i_*), and average summer stream temperature (*T*) to estimate the excretion rates of each individual sampled in our survey of Fox Cr. for both N and P. Excretion rates (N, P) for each individual were converted to daily rates (per 24 hr.) and summed across all individuals (n = 527 *O. mykiss*) to estimate the total amount and ratio of N and P recycled by *O. mykiss* in the 1 km study reach. In freshwater teleosts, urea and ammonium generally constitute the majority of the total nitrogenous end-products of metabolism [65], and in *O. mykiss* specifically, the greatest portion is excreted as ammonium [66], [67]. To directly compare *O. mykiss* excretion to *D. tenebrosus* excretion (NH_4_), we considered in our results only the portion of total excreted nitrogen by *O. mykiss* that can be attributed to ammonium (59.3%, 3.99 SE in *O. mykiss*; [67]). Ammonium also constitutes the bulk of nitrogenous excretion in aquatic amphibians [68]–[70]. We therefore consider ammonium as a conservative proxy for total N excretion in this study. It is generally established that SRP constitutes the bulk of excreted P, and can therefore be considered an acceptable estimate of total excreted P (e.g. [20], [24], [71]).

**Table 1:** Average elemental body composition (by dry mass) of common *O. mykiss* and *D. tenebrosus* diet items* by order.

			% elemental composition (dry mass±SD)		
Order	% of *O. mykiss* diet	% of *D. tenebrosus* diet	C	N	P
Coleoptera	9.25	0.50	53.6±1.0	8.7±0.7	0.5±0.1
Diptera	8.82	0.25	44.7±2.9	10.5±1.1	1.5±0.1
Ephemeroptera	26.82	75.53	46.2±4.2	10.7±1.0	1.2±0.2
Hemiptera^1^	0.45	1.15	50.7±4.2	11.7±0.9	1.0±0.3
Hymenoptera^2,3^	10.06	0.03	39.1± –	10.4± –	0.9± –
Lepidoptera^4,5^	6.24	1.69	34.6± –	5.8± –	0.3± –
Odonata	–	0.59	45.0±2.4	12.3±0.3	1.0±0.1
Orthoptera	–	0.54	46.8±1.7	9.6±0.3	0.7±0.1
Plecoptera^6^	0.37	0.85	52.9±0.4	10.3±0.2	1.1±0.1
Salmonid	–	6.93	43.6±7.0	12.7±2.3	1.3±0.2
Trichoptera	5.11	1.40	47.3±2.8	9.7±1.3	1.1±0.3

Elemental composition estimates from the literature for orders Lepidoptera and Hymenoptera did not include estimates of variability.

Footnotes: *Unaccounted for percentage of diet was comprised of diet items not covered by invertebrate CNP survey and for which values could not be found in literature. Contributions by these uncommon items were deemed inconsequential due to their small individual proportion of the wet mass of diets. Large and/or unique diet items (orders comprising <0.5% of total items) were discounted in diets so as not to bias elemental estimates.

1 Frost PC, Tank SE, Turner MA, Elser JJ (2010) Elemental composition of littoral invertebrates from oligotrophic and eutrophic Canadian lakes. Journal of the North American Benthological Society 22:51–62.

2 Elser JJ (2003) Biological stoichiometry: a theoretical framework connecting ecosystem ecology, evolution, and biochemistry for application in astrobiology. International Journal of Astrobiology 2:185–193.

3 Woods HA, Fagan WF, and Elser JJ (2004) Allometric and phylogenetic variation in insect phosphorus content. Functional Ecology 18:103–108.

4 Elser JJ, Fagan FF, Denno RF, Dobberfuhl DR, Folarin A, Huberty A, Interlandi S, Kilham SS, McCauley E, Schulz KL, Siemann EH, Sterner RW (2000) Nutritional constraints in terrestrial and freshwater food-webs. Nature 408:578–580.

5 Slansky Jr. F, and Feeny P (1977). Stabilization of the rate of nitrogen accumulation by larvae of the cabbage butterfly on wild and cultivated food plants. Ecological Monographs 47:209–228.

6 Cross WF, Benstead JP, Rosemond AD, and Wallace JB (2003) Consumer-resource stoichiometry in detritus-based streams. Ecology Letters 6:721–732.

### Ecosystem Nutrient Recycling

By combining measured excretion rates of *D. tenebrosus*, with bioenergetics estimates for *O. mykiss*, we predicted the total amount and ratios of N and P recycled by these two predators across the 1 km study reach. We performed a bootstrap numerical simulation [72] on the data using R (version 2.12.1) to provide a mean and a 95% confidence interval for the estimate of total excreted nitrogen and phosphorus for each species. In each bootstrap simulation (n = 10,000), individuals of each species from our survey dataset were randomly selected with replacement until the summed biomass of selected individual (*M_t-sim_*) equaled that observed for each species in our field survey of Fox Cr. for the study reach (*O. mykiss M_t-obs_* = 1733 g wet mass, *D. tenebrosus M_t-obs_ = *4542 g wet mass). Mass specific nutrient excretion rates were estimated for each selected individual as above, and summed to estimate the species-specific total N and total P recycled, as well as the N to P ratio. Each individual’s mass-specific excretion rates were estimated including parameter variability, as reported in the literature bioenergetics model in the case of *O. mykiss*, and from our empirically derived regressions in the case of *D. tenebrosus*. To assess the importance of the relative abundance of the two predators for nutrient recycling, we also ran simulations that varied the proportion of the predator biomass made up by salamanders and fish, assuming that the combined predator biomass we observed in Fox Cr. is a conservative estimate of the maximum sustainable regardless of predator identity. As such, we varied the predator composition from 100% *O. mykiss* to 100% *D. tenebrosus* biomass in 10% increments (n = 1,000 simulations for each increment).

To evaluate the magnitude of nutrient recycling by top predators relative to ecosystem demand, we calculated rates of community nutrient uptake in the study reach. Areal uptake rates were calculated based on Schade *et al*. [61] to provide estimates of nutrient demand during the summer growth period. Uptake rate (*U*: µg⋅m^−2^ s^−1^) can be calculated by multiplying discharge rate (*Q*: Ls^−1^) by nutrient concentration (*C*: µg⋅L^−1^), and dividing by reach width (*w*: m) and nutrient-specific spiral length (*S_w_*: m).
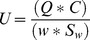



The model was parameterized using measurements from 32 replicated pools (*w, Q*) measurements combined with data presented by Schade *et al.* (*S_wN_*_:_ 200,*S_wP_*: 540, *C_N_*: 10.1, *C_P_*: 15) [61]. Aerial uptake rates (*U*) can be used to estimate the proportion of nutrient demand supplied by *O. mykiss* and *D. tenebrosus* excretion in the study reach by difference. These estimates consider the largest single component of dissolved inorganic nitrogen (i.e. NH_4_) and total dissolved phosphorus (i.e. SRP), and so can thus be considered a conservative estimate of uptake demand. Proportion of demand supplied by *O. mykiss* and *D. tenebrosus* recycling was determined by dividing areal recycling rate (total recycling rate divided by total study area) by uptake rate (*U*).

## Results

### Abundance Survey

**Figure 1:**
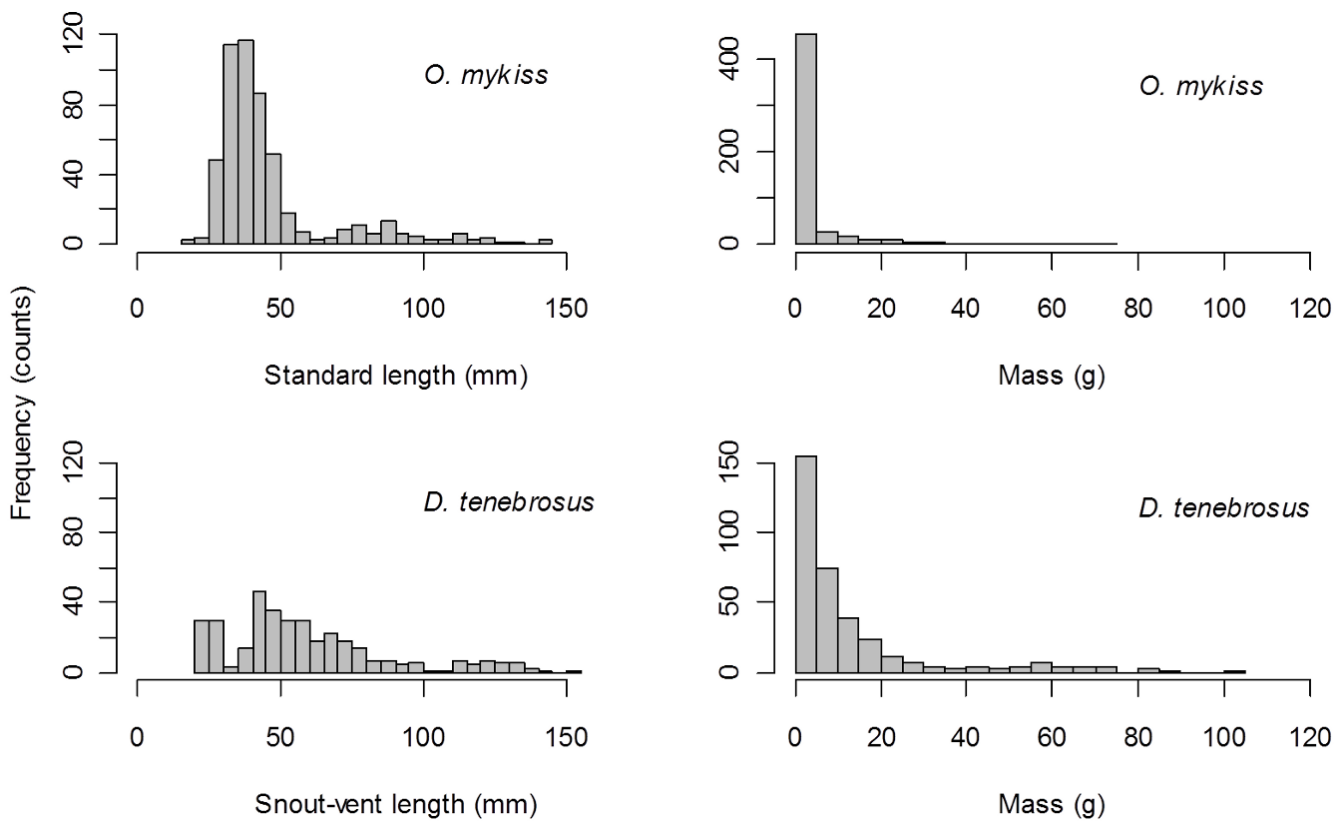
Histograms of size and mass of predators in our study reach. Size- and mass-frequency distributions for *O. mykiss* and *D. tenebrosus* from a 1 km study reach of Fox Creek. Standard length (mm) was used for fish (n = 528) and snout-vent length (mm) for salamanders (n = 348) to exclude the size variability generated by tail injuries. Note differences in y-axis scales.

During the field survey of the study area in Fox Cr., we captured 356 *D. tenebrosus* and 595 *O. mykiss*, at an average density of 1.05 and 1.76 individuals/m^2^ respectively (Table 2). Despite lower numerical abundance, the total biomass of *D. tenebrosus* was 2.5 times higher than *O. mykiss* (13.7 and 5.4 g wet mass/m^2^ respectively; Table 2). While the length range of captured individuals of each species was quite similar (Fig. 1, left panels), mean *D. tenebrosus* body mass was nearly 6 times higher (Fig. 1, right panels; *D. tenebrosus* = 13.05 g wet mass, 0.79 SE, *O. mykiss* = 3.28 g wet mass, 0.37 SE).

**Table 2:** Density and biomass (abundance survey) and average wet weight elemental composition of diets (diet survey) for *O. mykiss* and *D. tenebrosus* in Fox Creek, California.

	Abundance survey			Diet survey				
Species	Density (m^−2^)	Biomass (g*m^−2^)	*n*	% of diet accounted for	%C	%N	%P	*n*
*O. mykiss*	1.56	5.12	528	67.1	7.92	1.55	0.18	86
*D. tenebrosus*	1.03	13.42	348	89.5	8.51	1.88	0.23	55

Values calculated using total area of the study reach.

Our summary of invertebrate C, N, and P composition and predator diet composition showed that both species of predator had diets with a high degree of taxonomic overlap and similar elemental composition. Both *O. mykiss* and *D. tenebrosus* diets were dominated by Order Ephemeroptera (>25% by mass; Table 1). Carbon (min = 34.6%, max = 53.6% dry mass), N (min = 5.8%, max = 12.7% dry mass), and P (min = 0.3%, max = 1.5% dry mass) content varied substantially among orders (Table 1), and in total our analysis accounted for 90% of *D. tenebrosus* and 67% of *O. mykiss* diet items by mass (Table 1). The majority of unaccounted-for diet was composed of unidentifiable remains. We found that on average, *D. tenebrosus* diets were 8.5% C, 1.9% N, and 0.2% P by wet mass, and *O. mykiss* diets were composed of 7.9% C, 1.6% N, and 0.2% P by wet mass (Table 1). Only a small percentage of the salamanders surveyed had empty stomachs (5%), which is similar to the 1% occurrence of empty stomachs reported by Parker [45], which served as the basis for our decision to feed excretion trial salamanders *ad libitum*.

### Quantification of Excreted Nutrients

#### *D. tenebrosus* field study

**Figure 2:**
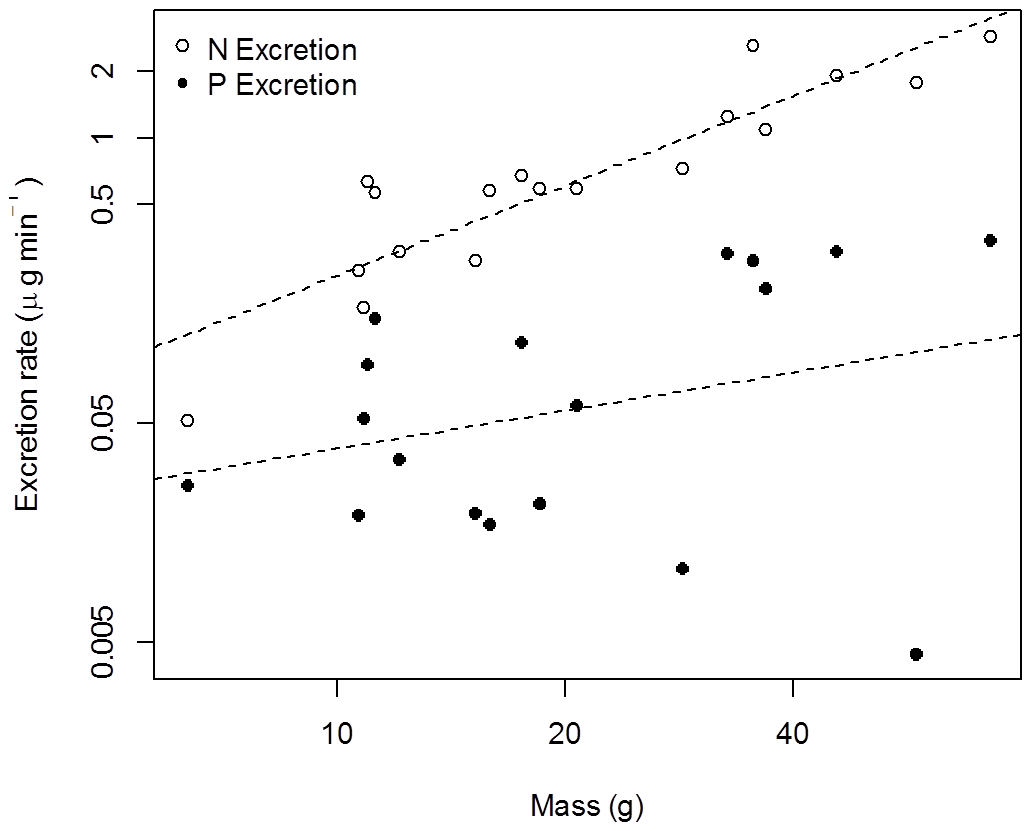
Excretion rates of *D. tenebrosus*. Nitrogen (NH_4_) and phosphorus (SRP) nutrient excretion rates (ug·min^−1^) of *D. tenebrosus*. Lines represent the fit of the top model selected by AICc for P (*log_10_*[µg_P_·min^−1^] = −3.12+1.60(*log*_10_[mass]), r^2^ = 0.31, P = 0.01), and N excretion rates (*log_10_*[µg_N_·min^−1^] = −2.04+1.41(*log*_10_[mass]), r^2^ = 0.79, P<<0.001).

Control containers showed no significant change in nutrient concentration over time (N: t = 0.56, df = 2, p-value = 0.63, P: t = 0.10, df = 2, p-value = 0.93) indicating that any changes we observed in trials containing salamanders were attributable to animal excretion, and not microbial processes. Model selection by AICc of N and P excretion rates for *D. tenebrosus* from our experiment identified clear top models for both nutrients, with ΔAICc scores of all other models >3 (Table S2). The best-supported model for *D. tenebrosus* P (SRP) excretion rate included both intercept and mass (*log_10_*[µg_P_·min^−1^] = −3.12+1.60·*log_10_*[mass], SE_intercept_ = 0.73, SE_mass coefficient_ = 0.56; Fig. 2), as did the best-supported model for N (NH_4_) excretion rate (*log_10_*[µg_N_·min^−1^] = −2.04+1.41·*log_10_*[mass], SE_intercept_ = 0.24, SE_mass_ _coefficient_ = 0.18; Fig. 2). We found little support for models that included diet treatment (terrestrial invertebrate, aquatic invertebrate, and aquatic vertebrate), where all models including diet had ΔAIC ≥3. Consequently, dietary treatment was disregarded for *D. tenebrosus* in subsequent analyses.

#### *O. mykiss* bioenergetics model

Results from the diet survey and the invertebrate body C, N, and P survey were used to establish the N and P composition of an average steelhead diet (Table S3; N = 1.8%, P = 0.2% wet weight). Diet information was combined with average stream temperature (14.2°C, 0.004 SE, min = 12.0°C, max = 16.5°C) in our bioenergetics model. Using these inputs, rate of excretion of N and P was calculated for each individual and used for the ecosystem analysis.

### Ecosystem Nutrient Recycling

**Figure 3:**
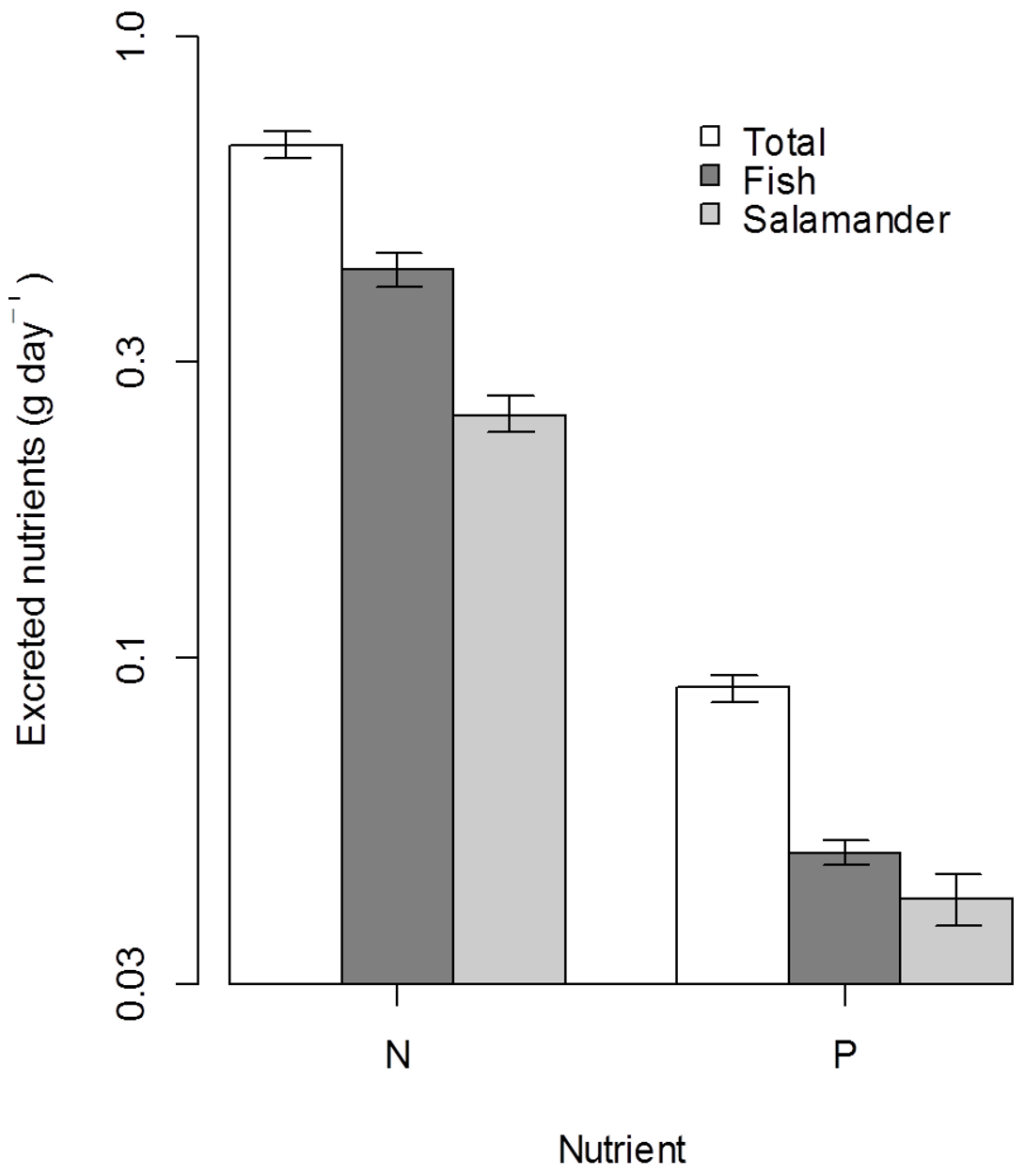
Daily excretion estimates for predators in our study reach. Estimated total daily excreted N (NH_4_) and P (SRP) by *O. mykiss* (filled), *D. tenebrosus* (grey), and both predators combined (open) within the Fox Cr. study reach. Bars represent mean ±95%CI. Note the log scaled y-axis.

**Figure 4:**
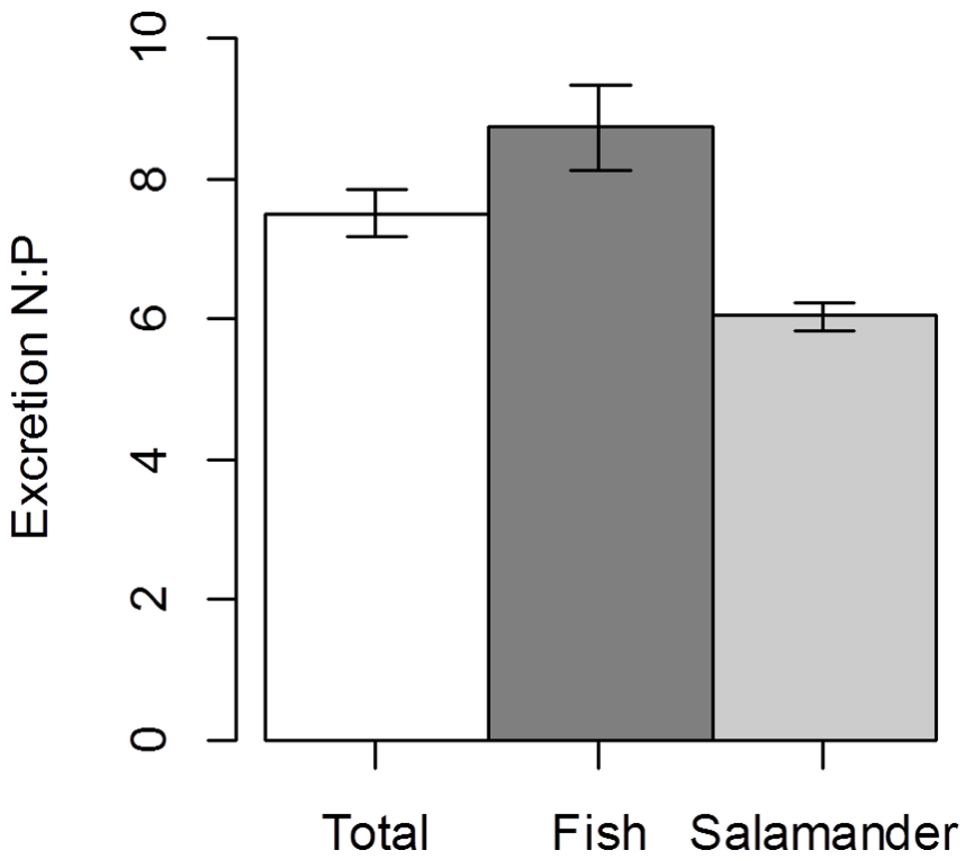
Estimates of excreted N:P ratio for predators in our study reach. Estimates of the ratio of excreted N:P for *O. mykiss* (filled), *D. tenebrosus* (grey), and both predators combined (open). Bars represent mean ±95%CI.

We estimated the daily N and P recycled by individual *D. tenebrosus* and *O. mykiss*, by species, and by both species combined within the study reach. We estimated that *O. mykiss* recycled nearly twice as much N per day (0.42 g·day^−1^±0.028 95% CI) as that recycled by *D. tenebrosus* (0.25 g·day^−1^±0.017 95% CI, Fig. 3) within the study reach. The phosphorus recycled by *O. mykiss* (0.049 g·day^−1^±0.0021 95% CI) was approximately 19% greater than that recycled by *D. tenebrosus* (0.041 g·day^−1^±0.0039 95% CI). We found that when estimates for both species were summed, 0.67 g·day^−1^ of N was recycled (±0.032 95% CI, Fig. 3A), and 0.089 g day^−1^ of P (±0.0044 95% CI). The N:P ratio of nutrients recycled by *O. mykiss* (8.7±0.6 95% CI, Fig. 4) was higher than that of *D. tenebrosus* (6.0±0.2 95% CI) and the N:P ratio of both species combined (7.5±0.3 95% CI) was intermediate between the two.

**Figure 5:**
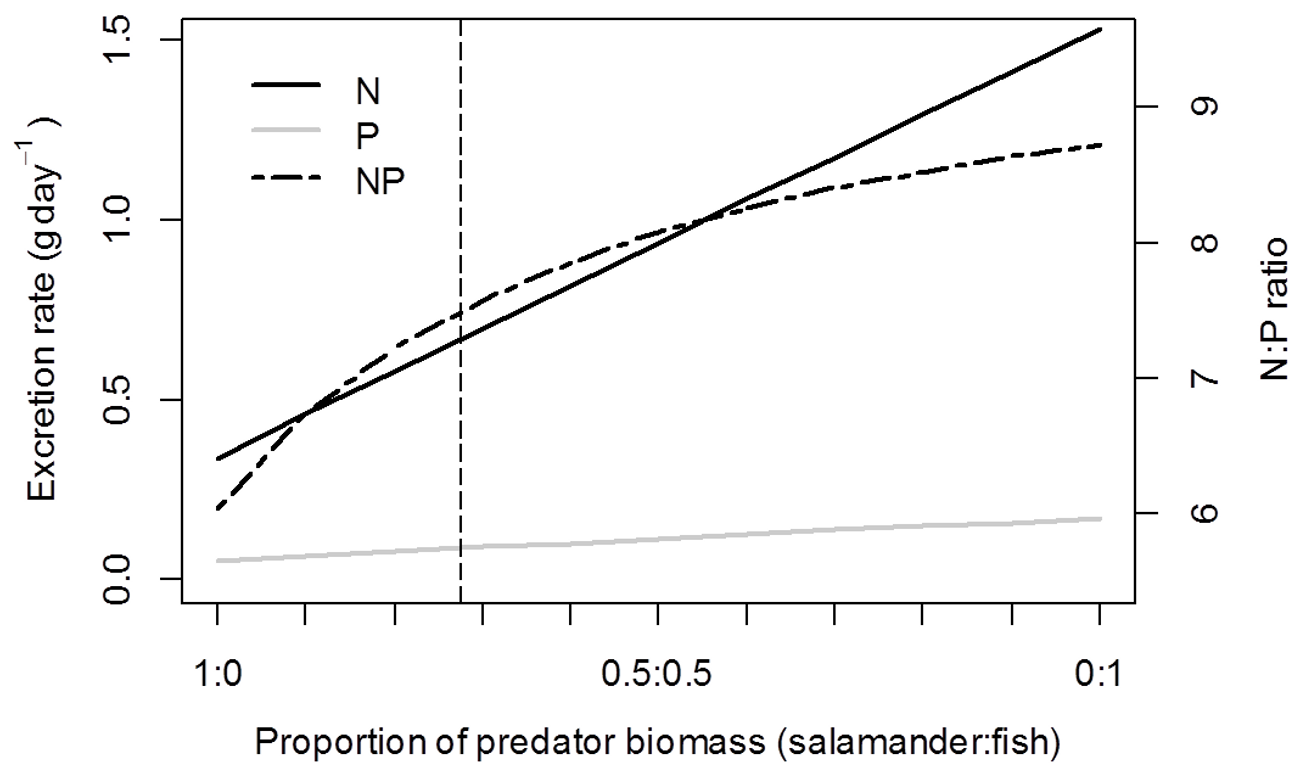
Impacts on excretion of simulated tradeoffs between predator biomasses. Estimated total recycled N (NH_4_) and P (SRP) excretion (g·day^−1^) in Fox Cr. due to simulated changes in the relative abundance (by biomass) of *O. mykiss* and *D. tenebrosus*. Simulations assumed a fixed total biomass of predators (6275 g) within the study reach, and estimated total excretion rates (left y-axis) and ratios (right y-axis) by bootstrapped re-sampling of surveyed individuals. Predator relative abundance (x-axis) varies by 10% increments from 100% *O. mykiss* composition to 100% *D. tenebrosus* composition, expressed as the proportion of predator biomass (salamander:fish). Vertical line indicates the observed ratio of predators in Fox Cr.

We found that varying the relative abundance of the two vertebrate predators (assuming constant total biomass) led to large differences in total excreted nutrients (N and P). When we simulated the predator biomass as being entirely comprised of *D. tenebrosus*, N and P excretion rates were estimated to be 0.34 and 0.06 g·day^−1^ respectively (Fig. 5). These rates increased along the relative abundance continuum to a maximum N and P excretion rate of 1.53 and 0.18 g·day^−1^ respectively when *O. mykiss* comprised all the predator biomass. The overall N:P ratio of predator-recycled nutrients ranged from 6.04 when all biomass was *D. tenebrosus*, to 8.72 when all biomass was *O. mykiss* (Fig. 5).

We calculated that uptake rates (*U*) for N and P were 0.1 µg·m^−2^ s^−1^ and 0.04 µg·m^−2^ s^−1^ respectively. At the observed densities of *D. tenebrosus* and *O. mykiss*, we estimated that nutrient recycling by predators in the study reach represented 20.3% of N (0.0023 µg·m^−2^ s^−1^±1.1*10^−4^ 95% CI) and 7.3% of P (3.1*10^−4^ µg·m^−2^ s^−1^±1.5*10^−5^ 95% CI) demand by producers in the study reach.

## Discussion

This study suggests that differences in predator in abundance, body size distribution, and species identity directly influence nutrient recycling rates and ratios in headwater streams. These findings emphasize the importance of species identity in predator-driven nutrient recycling. As such, we found that biomass alone is not an adequate proxy for estimating species contributions to the total rate of nutrient recycling, which depends on physical, physiological, and ecological traits of different members of the predator guild. Despite more than two and a half times higher biomass, the total amount of nutrients recycled by *D. tenebrosus* in Fox Cr. was only a fraction (58% N, 84% P) of that recycled by *O. mykiss*. Despite a lower relative contribution, the *D. tenebrosus* population in Fox Cr. did provide 37% of the total N and 46% of the total P recycled by vertebrate predators, providing support for the importance of vertebrates other than fish in stream nutrient recycling. We also found that the relative abundance of salamander and fish biomass altered the total amount of nutrients recycled by top predators per day. Our simulations suggest that when predator biomass is comprised completely of *D. tenebrosus*, the amount of recycled N and P are only approximately half (51% N, 63% P relative to background) that recycled by the natural assemblage of salamanders and fish in Fox Cr. When the total predator biomass in our simulation was comprised entirely of *O. mykiss*, both N and P recycling rates were estimated to be approximately twice as high as background (2.3×N, 2.0×P).

The large differences we found in estimated stream-wide excretion rates between *O. mykiss* and *D. tenebrosus* are in contrast to the many ecological similarities we found between the species. As ambush predators, *D. tenebrosus* feed mostly on aquatic benthic prey [73], whereas *O. mykiss* feed throughout the water column, and consume a larger proportion of allochthonous (e.g. terrestrial) prey. While each predator feeds in different stream microhabitats, they have a high degree of overlap in diet composition (Table 1, also see [44]), with similar elemental composition. Our data suggest that dietary or stoichiometric differences in prey are not likely to explain the observed differences in species-specific N:P ratios (Table S3). Similarly, data from a complementary study found that growth rates of *D. tenebrosus* and *O. mykiss* in the same study reach did not differ significantly over the summer growth period (Atlas et al., *unpublished data*), indicating that differences we identified in nutrient excretion rates are likely driven by differences in metabolic requirements. At zero activity level, *O. mykiss* is estimated to have a 50% higher metabolic demand as measured by oxygen consumption [74] than an average salamander of the same weight [75]. *D. tenebrosus* is a sit-and-wait predator and is only marginally active at night and even less so during the day, whereas *O. mykiss* is an active water-column predator and expends large amounts of energy for locomotion and foraging when not resting or seeking cover in interstitial spaces [60]. Larger expenditures of energy by *O. mykiss* likely necessitate higher metabolic rates and faster production of metabolic wastes, which is excreted at a higher mass-specific rate than by *D. tenebrosus*. An additional factor that likely contributes to the difference in excretion ratios is species specific elemental requirements for growth and maintenance. Stoichiometric theory suggests that as the required N:P ratio of an organism increases at any given ingested N:P ratio, the ratio of excreted nutrients will decrease [76]. Though we do not have elemental composition data for *D. tenebrosus* from our study system, values from the literature show that eastern salamanders that fill a similar ecological niche have organismal N:P ratios of 2.4–4.8 [36], whereas juvenile steelhead in our study system had an average N:P ratio of 9.8. However, our findings that *O. mykiss* recycles N:P at a higher ratio than *D. tenebrosus* suggests that the body N:P ratio of *D. tenebrosus* may be much higher than eastern species.

Metabolic scaling theory predicts that as mass increases, mass specific excretion rate should decrease [77], which may further explain the disparity in total excreted nutrients between predator species. The higher numbers but lower biomass of *O. mykiss* as compared to *D. tenebrosus* (Table 2), resulted from a smaller average size of *O. mykiss* individuals in our study system (mean O. mykiss = 3.3 g wet mass, mean D. tenebrosus = 13.1 g wet mass). The size-frequency distribution of *O. mykiss* (Fig. 1), illustrates two distinct nodes representing young of the year (age 0) and one-year or older age-classes that make up the vast majority of *O. mykiss* individuals. This distribution is typical of populations consisting of primarily anadromous individuals that migrate out of freshwaters after approximately 1.5 years (summarized by [78]), and is also indicative of the consistent growth pattern exhibited by most salmonids [79]–[81]. By contrast, there is a near continuous distribution of *D. tenebrosus* body size, with no distinct nodes and a higher frequency of larger individuals than *O. mykiss* (Fig. 1). Coastal giant salamanders can achieve life spans in excess of 20 years, and are characterized by slow growth, leading to difficulty in assigning age based on body size. Ultimately, the larger average mass achieved by *D. tenebrosus* compared to *O. mykiss* is likely to contribute to the observed lower mass-specific excretion rate and lower total species-specific rate of nutrient recycling.

Our test of the influence of diet on excretion ratios in *D. tenebrosus* showed that despite the differing N:P ratios of the items fed to captive individuals (aquatic invertebrate = 12.7, terrestrial invertebrate = 13.4, aquatic invertebrate = 9.9), we found no support for differences in N or P excretion rates of *D. tenebrosus* fed these prey. Our results were likely inconsistent with our stoichiometric prediction for several reasons. Despite holding salamanders with food items for a full iteration of their estimated digestive throughput time (60 hours), individual excretion rates may not have had time to equilibrate to reflect the homogenous experimental diets. Additional factors may have included a wide range of body sizes (6.3–72.8 mm SVL), individuals at different stages of reproductive maturity [82], and small sample size (*n* = 18).Our findings demonstrate that vertebrate excretion can affect nutrient availability in light-limited forested streams. However, the repercussions of altering nutrient availability depend on the nutrient requirements of producers in the system. By increasing the availability of limiting nutrients, algal growth and elemental composition can be altered. Using nutrient addition experiments, Schade *et al.* [61] demonstrate that Fox Cr. is N-limited, and has an external dissolved inorganic NH_4_-N:SRP ratio of 0.67. The ratio of recycled nutrients from predators examined in this study is modestly higher than ambient levels (7.5; Fig. 3), suggesting the potential for partial alleviation of the N-limitation experienced by autotrophs in Fox Cr. Due to the higher ambient concentrations of inorganic nutrients in Fox Cr. compared to other similar tributary streams [61], it is likely that the contribution by predators to nutrient availability in other streams that support similar predator densities is greater than in Fox Creek. This suggests that our estimates of the percent of nutrient demand supplied by vertebrates in Fox Cr. may be lower than other streams in the region.

River networks are characterized by strong downstream gradients in light, temperature, disturbance frequency, and physical channel attributes, each of which can differentially affect the movement, growth, and persistence of riverine species [83], [84]. Despite having highly overlapping regional distributions, *O. mykiss* and *D. tenebrosus* vary in their relative abundance across the heterogeneity present in river networks at local-scales [40]. Our simulation results suggest that such differences, combined with spatial and temporal variation in ecosystem nutrient demand, are likely to affect broad-scale patterns of nutrient limitation. While the study system we worked in is within a protected area, and has been well buffered from local anthropogenic impacts, the relative abundance of these two species across broader watersheds and larger coastal regions is likely affected by species-specific responses to anthropogenic stressors in the past as well as the future. In the past, steelhead abundance was very high along the entire west coast of North America, but overexploitation, especially along the California coastline, has drastically diminished abundance [85]. Future thermally limiting events may also affect abundances. Salmonids are known to have much lower critical thermal maxima, both as juveniles (26.2–27.9°C; [86]) and older classes (28.25–29.85; [87]), than many salamanders (30.1–37.3°C; [88]). As summer maximum temperatures increase with future climate change and continued land-cover change (e.g. forest clearing), amphibian populations may be able to persist in thermal environments that cause acute mortality or exclude salmonid populations. Our study suggests that in such cases, large salamander populations may act to partially buffer the impacts of declining fish populations via nutrient recycling. By not considering amphibians and other animals as nutrient recyclers, we may be missing critical components of nutrient dynamics in stream ecosystems.

This study provides an estimate of excretion rates for a dominant stream predator in the Pacific Northwest that has not been previously studied. The bulk of literature in the field has examined highly productive tropical streams or lentic ecosystems (e.g. [15], [25], [29], [30], [89]). We show here that nutrient recycling by top predators is an important component of ecosystem nutrient dynamics even in small temperate streams. Though amphibians have been previously considered, their importance as nutrient recyclers has been discounted due to low densities compared to other vertebrate taxa (but see [35], [90]). Salamanders in many stream ecosystems have strong top-down effects on prey abundance [91]–[93], and act as sources of energy for higher level predators [36], [94], [95]. This study demonstrates that amphibians can also contribute to ecosystem-level dynamics through recycling of nutrients, in this case accounting for 37% of N and 46% of P recycled by vertebrates. Our findings suggest that studies of consumer-driven nutrient recycling will continue to benefit by identifying additional key recyclers in various systems. Continued work in this field will help expand our knowledge of the ecological roles of many organisms, and foster a better understanding of nutrient dynamics in aquatic systems.

## Supporting Information

Table S1 **Sources for invertebrate length-weight regression used to determine the mass of individual insects for the diet survey.**
(DOCX)

Table S2 **AICc scores for models predicting N and P excretion rates.**
(DOCX)

Table S3 **Raw excretion data from all** ***Dicamptodon tenebrosus*** **individuals used in the excretion study.** Excretion rates were measured at t = 0 min and t = 120 min. Diet treatment codes are as follows: AM: Aquatic macroinvertebrate, YOY: young-of-the-year steelhead, TM: Terrestrial macroinvertebrate.
(DOCX)
