# Supplementary material for: Predator-Driven Nutrient Recycling in California Stream Ecosystems
Source: PLoS One. 2013 Mar 8;8(3):e58542. doi: 10.1371/journal.pone.0058542 (PMC3592796; doi:10.1371/journal.pone.0058542)
Supplement: Table S1 — Sources for invertebrate length-weight regression used to determine the mass of individual insects for the diet survey. (DOCX) [file pone.0058542.s001.docx]

**Table S1**

| **Order** | **Taxon** | **Source** |
| --- | --- | --- |
| Acari | Mite (Adult) | Hodar (1996) |
| Aranaea | Spider (Adult) | Sabo *et al*. (2002) |
| Coleoptera | Ciidae (Adult) | Sabo *et al*. (2002) |
|  | Coleoptera (General adult) | Sabo *et al*. (2002) |
|  | Coleoptera (General larva) | Hodar (1996) |
|  | Curculionidae (Adult) | Hodar (1996) |
|  | Psephenidae (Larva) | Benke *et al.*(1999) |
|  | Staphylinidae (Adult) | Sabo *et al*. (2002) |
| Collembola | Collembola (General adult) | Hodar (1996), Ganihar (1997) |
| Diptera | Brachycera (Adult) | Sample *et al.* (1993) |
|  | Brachycera (Larva) | Benke *et al.*(1999) |
|  | Chironomidae | Benke *et al.*(1999) |
|  | Diptera (General adult) | Hodar (1996) |
|  | Diptera (General larva) | Benke, *et al.*(1999) |
|  | Dixidae (Larva) | Benke, *et al.*(1999) |
|  | Nematocera (Adult) | Sample, *et al.* (1993) |
|  | Simuliidae (Larva) | Benke, *et al.*(1999) |
| Ephemeroptera | Ameletidae | Benke, *et al.*(1999) |
|  | Baetidae (Nymph) | Benke, *et al.*(1999) |
|  | Ephemeroptera (Nymph) | Benke, *et al.*(1999) |
|  | Heptageneiidae (Nymph) | Benke, *et al.*(1999) |
|  | Paraleptophlebia (Nymph) | (developed in lab) |
| Hemiptera | Cicadidae (Adult) | Sabo, *et al*. (2002) |
|  | Hemiptera (Adult) | Schoener (1980), Sample, *et al*. (1993), Hodar (1996), Ganihar (1997), Rogers, *et al.* (1976) |
|  | Veliidae | Benke, *et al.*(1999) |
| Hymenoptera | Formicidae (Adult) | Sabo, *et al*. (2002) |
|  | Hymenoptera (Adult) | Rogers, *et al.* (1976), Schoener (1980), Sample, *et al*. (1993), Ganihar (1997) |
| Isopoda | Isopoda (General adult) | Hodar (1996) |
| Lepidoptera | Lepidoptera (Larva) | Hodar (1996) |
| Megaloptera | Orohermes (Larva) | Benke, *et al.*(1999) |
| Odonata | Cordulegastridae (Nymph) | Benke, *et al.*(1999) |
|  | Gomphidae (Nymph) | Sabo, *et al*. (2002) |
| Orthoptera | Orthoptera (General adult) | Sabo, *et al*. (2002) |
| Plecoptera | Calineuria | Benke, *et al.*(1999) |
|  | Hesperoperla (Nymph) | Benke, *et al.*(1999) |
|  | Nemouridae (Nymph) | Benke, *et al.*(1999) |
|  | Perlidae (Nymph) | Benke, *et al.*(1999) |
| Salmoniformes | Steelhead | Boldt and Haldorson (2002) |
| Trichoptera | Brachycentridae | Benke, *et al.*(1999) |
|  | Glossosoma (Larva) | Benke, *et al.*(1999) |
|  | Gumaga (Nymph) | Benke, *et al.*(1999) |
|  | Lepidostoma (Larva) | Benke, *et al.*(1999) |
|  | Limnephilidae (Larva) | Benke, *et al.*(1999) |
|  | Neophylax (Larva) | (developed in lab) |
|  | Parapsyche (Larva) | Benke, *et al.*(1999) |
|  | Philopotamidae (Larva) | Benke, *et al.*(1999) |
|  | Polycentropotidae (Larva) | Benke, *et al.*(1999) |
|  | Rhyacophilidae (Larva) | Benke, *et al.*(1999) |
|  | Trichoptera (General adult) | Sabo, *et al*. (2002) |
|  | Trichoptera (General nymph) | Benke, *et al.*(1999) |
|  | Trichoptera (General pupae) | Benke, *et al.*(1999) |
| Unknown | Unknown (Not fish) | Rogers, *et al.* (1976) |

Benke, A. C., A. D. Huryn, L. A. Smock, and J. B. Wallace (1999). Length-Mass Relationships for Freshwater Macroinvertebrates in North America with Particular Reference to the Southeastern United States. *Journal of the North American Benthological Society* 18:308-343.

Boldt, J.L and L.T. Haldorson (2002). A bioenergentics approach to estimating consumption of zooplankton by juvenile pink salmon in Prince William Sound, Alaska. *Alaska Fisheries Research Bulletin* 9:111-127.

Ganihar, S. R. (1997). Biomass estimates of terrestrial arthropods based on body length. *Journal of Biosciences* 22:219-224.
Hodar, J.A. (1996). The use of regression equations for estimation of arthropod biomass in ecological studies. Acta Oecologica-International Journal of Ecology. 17:421-433.

Rogers, L. E., W. T. Hinds, and R. L. Bushbom (1976). A general weight vs. length relationship for insects. Annals of *the Entomological Society of America* 69:387-389

Sabo, J. L., J. L. Bastow, and M. E. Power (2002) Length–mass relationships for adult aquatic and terrestrial invertebrates in a California watershed. *Journal of the North American Benthological Society* 21:336-343.

Sample, B. E., R. J. Cooper, R. D. Greer, and R. C. Whitmore (1993). Estimation of insect biomass by length and width. *American Midland Naturalist* 129:234-240.

Schoener, T. W. (1980). Length-weight regressions in tropical and temperate forest-understory insects. *Annals of the Entomological Society of America* 73:106-109.
