# Supplementary material for: Predator-Driven Nutrient Recycling in California Stream Ecosystems
Source: PLoS One. 2013 Mar 8;8(3):e58542. doi: 10.1371/journal.pone.0058542 (PMC3592796; doi:10.1371/journal.pone.0058542)
Supplement: Table S2 — AICc scores for models predicting N and P excretion rates. (DOCX) [file pone.0058542.s002.docx]

**Table S2**

|  |  | ∆AICc Score | |
| --- | --- | --- | --- |
| Model |  | N | P |
| 1 | ln(excretion rate) ~ 1 | 24.7 | 4.4 |
| 2 | ln(excretion rate) ~ ln(mass) | 0 | 0 |
| 3 | ln(excretion rate) ~ treatment | 24.4 | 10.3 |
| 4 | ln(excretion rate) ~ ln(mass) + treatment | 2.9 | 7 |
| 5 | ln(excretion rate) ~ ln(mass)*treatment | 10.8 | 13.5 |
