# Supplementary material for: Predator-Driven Nutrient Recycling in California Stream Ecosystems
Source: PLoS One. 2013 Mar 8;8(3):e58542. doi: 10.1371/journal.pone.0058542 (PMC3592796; doi:10.1371/journal.pone.0058542)
Supplement: Table S3 — Raw excretion data from all Dicamptodon tenebrosus individuals used in the excretion study. Excretion rates were measured at t = 0 min and t = 120 min. Diet treatment codes are as follows: AM: Aquatic macroinvertebrate, YOY: young-of-the-year steelhead, TM: Terrestrial macroinvertebrate. (DOCX) [file pone.0058542.s003.docx]

**Table S3**

| **Animal #** | **SVL (mm)** | **Mass (g)** | **Treatment** | **Excretion P**  **(ug/min/g_salamander_)** | **Excretion N**  **(ug/min/ g_salamander_)** | **N:P** |
| --- | --- | --- | --- | --- | --- | --- |
| RM-049 | 115 | 45.5 | AM | 0.0067 | 0.0425 | 6.32 |
| RM-050 | 79 | 17.5 | AM | 0.0067 | 0.0385 | 5.71 |
| RM-074 | 97 | 28.6 | AM | 0.0004 | 0.0258 | 66.70 |
| RM-077 | 70 | 15.2 | AM | 0.0013 | 0.0182 | 14.07 |
| RM-182 | 63 | 10.6 | AM | 0.0018 | 0.0235 | 12.93 |
| RM-183 | 78 | 18.5 | AM | 0.0012 | 0.0317 | 27.19 |
| RM-051 | 67 | 10.9 | YOY | 0.0085 | 0.0590 | 6.92 |
| RM-056 | 133 | 72.8 | YOY | 0.0048 | 0.0404 | 8.50 |
| RM-075 | 107 | 35.3 | YOY | 0.0079 | 0.0754 | 9.60 |
| RM-076 | 97 | 32.7 | YOY | 0.0092 | 0.0387 | 4.19 |
| RM-185 | 75 | 15.9 | YOY | 0.0011 | 0.0362 | 32.72 |
| RM-186 | 115 | 58.1 | YOY | 0.0001 | 0.0311 | 404.47 |
| RM-052 | 74 | 11.2 | TM | 0.0135 | 0.0504 | 3.73 |
| RM-055 | 56 | 6.3 | TM | 0.0042 | 0.0083 | 1.98 |
| RM-078 | 67 | 12 | TM | 0.0029 | 0.0252 | 8.76 |
| RM-079 | 66 | 10.8 | TM | 0.0049 | 0.0159 | 3.24 |
| RM-187 | 104 | 36.8 | TM | 0.0056 | 0.0299 | 5.31 |
| RM-181 | 87 | 20.7 | TM | 0.0029 | 0.0284 | 9.74 |
